# Relation of Splanchnoptosis to Gastric Acidity

**Published:** 1915-11

**Authors:** 


**Relation of Splanchnoptosis to Gastric Acidity.** Edgar Poe Sandrock, Johns Hopkins Hosp. Bull., July 1915.

| | Lockwood 250 cases | Author 25 1st degree | Author 140 2d degree | Author 35 3d degree |
|---|---|---|---|---|
| Hyperchlorhydria | 27.4% | 28% | 12.8% | 25.7% |
| Euchlorhydria | 55.6 | 40 | 49.2 | 48.5 |
| Hypochlorhydria | 55.6 | 20 | 24.2 | 17.1 |
| Achylia | 9.5 | 12 | 13.5 | 8.5 |

By first degree, the author means cases in which the greater curvature is at the level of the pelvic brim; by second degree those in which it is below this level; and by third degree, those in which the stomach reaches the bottom of the pelvis. (Note: We candidly admit that if these terms are used in the gynaecologic sense and the level of the pelvic brim is taken anteriorly, even the first degree of ptosis is rare. We do not recall a case of the third degree. Standards of hydrochloric acidity are not definitely stated. Statements as to total acidity are also made but as the total acidity varies greatly from fermentation and hence usually though not regularly is inverse to hydrochloric acidity, these are not significant. It should be noted that while the proportion of achylic cases is very high, so high as to raise the question whether ferment-production was rigorously excluded, the statistics rather tend to prove that gastroptosis has nothing directly to do with secretion. There is no progressive tendency, according to degree of ptosis noted, except perhaps toward a normal condition. Generally speaking, the cases are assorted just about as they would be in any miscellaneous series, with the exception of achylia. Ptosis might a priori be expected to interfere with secretion of HCI, in either direction. While, of course, such an interference could not be expected to be absolute and thus to apply to all cases, it would be noted in some consistent way in the majority of all cases and it would naturally show an increase, in one way or the other, as more and more aggravated conditions are considered. The number of cases is sufficient to exclude individual error and the series seems, on the whole to corroborate the general clinical experience that ptosis has no direct and positive bearing on secretion).

